# Hyperosmotic tolerance of adult fish and early embryos are determined by discrete, single loci in the genus *Oryzias*

**DOI:** 10.1038/s41598-018-24621-7

**Published:** 2018-05-02

**Authors:** Taijun Myosho, Hideya Takahashi, Kento Yoshida, Tadashi Sato, Satoshi Hamaguchi, Tatsuya Sakamoto, Mitsuru Sakaizumi

**Affiliations:** 1 Laboratory of Molecular Reproductive Biology, Institute for Environmental Science, University of Shizuoka, Shizuoka, 422–8526 Japan; 2 Department of Environmental Science, Institute of Science and Technology, Niigata University, Ikarashi, Niigata, 950–2181 Japan; 3 Ushimado Marine Institute, Faculty of Science, Okayama University, Setouchi, Okayama, 701–4303 Japan

## Abstract

The acquisition of environmental osmolality tolerance traits in individuals and gametes is an important event in the evolution and diversification of organisms. Although teleost fish exhibit considerable intra- and interspecific variation in salinity tolerance, the genetic mechanisms underlying this trait remain unclear. *Oryzias celebensis* survives in sea and fresh water during both the embryonic and adult stages, whereas its close relative *Oryzias woworae* cannot survive in sea water at either stage. A linkage analysis using backcross progeny identified a single locus responsible for adult hyperosmotic tolerance on a fused chromosome that corresponds to *O*. *latipes* linkage groups (LGs) 6 and 23. Conversely, *O*. *woworae* eggs fertilised with *O*. *celebensis* sperm died in sea water at the cleavage stages, whereas *O*. *celebensis* eggs fertilised with *O*. *woworae* sperm developed normally, demonstrating that maternal factor(s) from *O*. *celebensis* are responsible for hyperosmotic tolerance during early development. A further linkage analysis using backcrossed females revealed a discrete single locus relating to the maternal hyperosmotic tolerance factor in a fused chromosomal region homologous to *O*. *latipes* LGs 17 and 19. These results indicate that a maternal factor governs embryonic hyperosmotic tolerance and maps to a locus distinct from that associated with adult hyperosmotic tolerance.

## Introduction

Adjusting to osmotically different environments is a major factor limiting the ability of freshwater organisms to colonize sea water, or for marine organisms to expand their distribution into freshwater systems. Elucidation of the genetic mechanisms underlying the physiological ability to tolerate osmotic challenges would provide insight into the evolutionary processes related to the alteration/expansion of a species’ habitat. Although several factors responsible for osmotic tolerance have been identified in plants and fungi^1–4^, there has been no significant progress in understanding how it is genetically determined in animals. It is well known that teleost species exhibit considerable intra- and interspecific variation in hyperosmotic/salinity tolerance, and it has been suggested that the ability of fish to adjust to sea water is modulated by genetic factors^5,6^. Furthermore, the transition from freshwater to seawater habitats requires that a species acquires hyperosmotic tolerance during certain life cycle stages, as is known in anadromous salmonids and catadromous eels. The gills (and the yolk sac) are the primary osmoregulatory organs where active extrusion of ions occur. In addition to the gills, major osmoregulatory sites are the gastrointestinal tract, which absorbs ions and water following drinking, particularly for acquisition of water, and the kidney, where divalent ions are excreted into sea water. However, the critical determinant of hyperosmotic tolerance and the genes underlying it have not been comprehensively studied. Previous studies have focused on these osmoregulatory organs and their specific genes, such as those for transporters^7^ and those observed in transcriptome changes during seawater challenges^8,9^.

**Figure 1:**
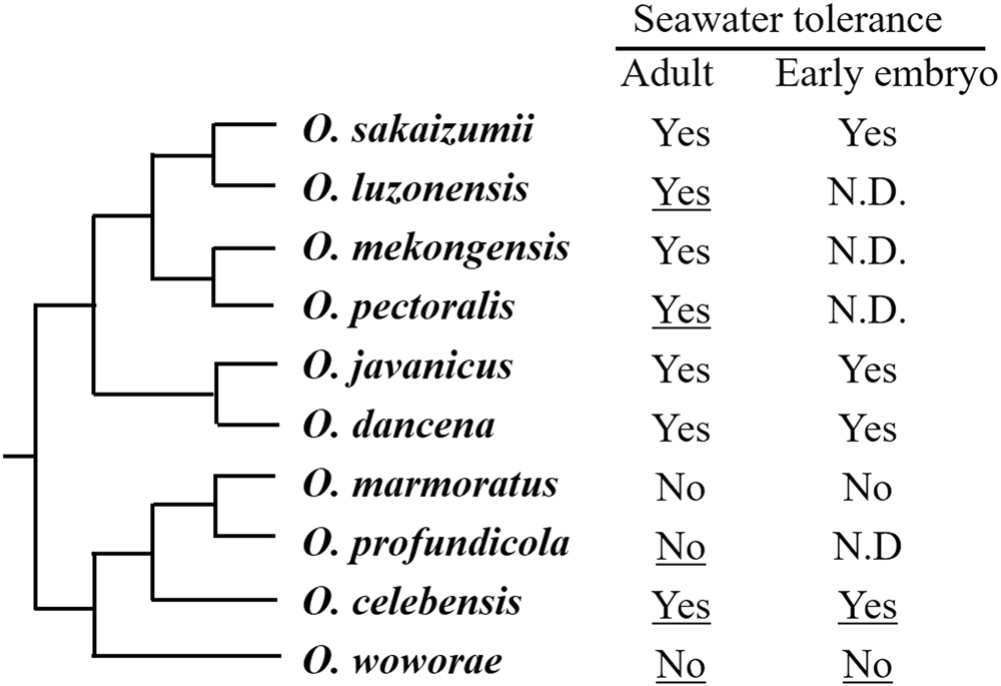
Phylogenetic relationships and seawater adjustability in *Oryzias* fishes showing that osmotic tolerance has evolved repeatedly. Phylogenetic information was taken from Takehana *et al*.^26^ and Mokodongan and Yamahira^27^. Species examined in the present study are underlined. “Yes” and “No” based on their ability to survive in fresh or sea water, respectively. N.D. indicates no data.

Medaka fishes in the genus *Oryzias* are frequently used as experimental animals for a variety of studies, including those in genetics and physiology. This genus consists of approximately 30 species, most of which inhabit freshwater environments, such as lakes, ponds, and paddy fields, and show diverse tolerance to salinity (e.g. brackish water and seawater)^10,11^. These factors suggest that species of *Oryzias* are potential models for studies of adjustment to varying salinities (Fig. 1).

In this study, we initially examined the hyperosmotic tolerance of several *Oryzias* species and found that two species in the *celebensis* group, *O*. *celebensis* and its close relative *O*. *woworae*, have markedly different adjustability to sea water in adult and early embryonic stages. To clarify the genetic basis of hyperosmotic tolerance, we conducted genetic linkage analyses using F_1_ and BC_1_ progeny derived from these species. Here, we reveal not only the locus of the genetic factor responsible for the hyperosmotic tolerance of adult fish, but also another locus of a maternal factor(s) for tolerance during the early embryonic stage.

## Results and Discussion

### Hyperosmotic tolerance of adult fish determined by a single major locus

**Figure 2:**
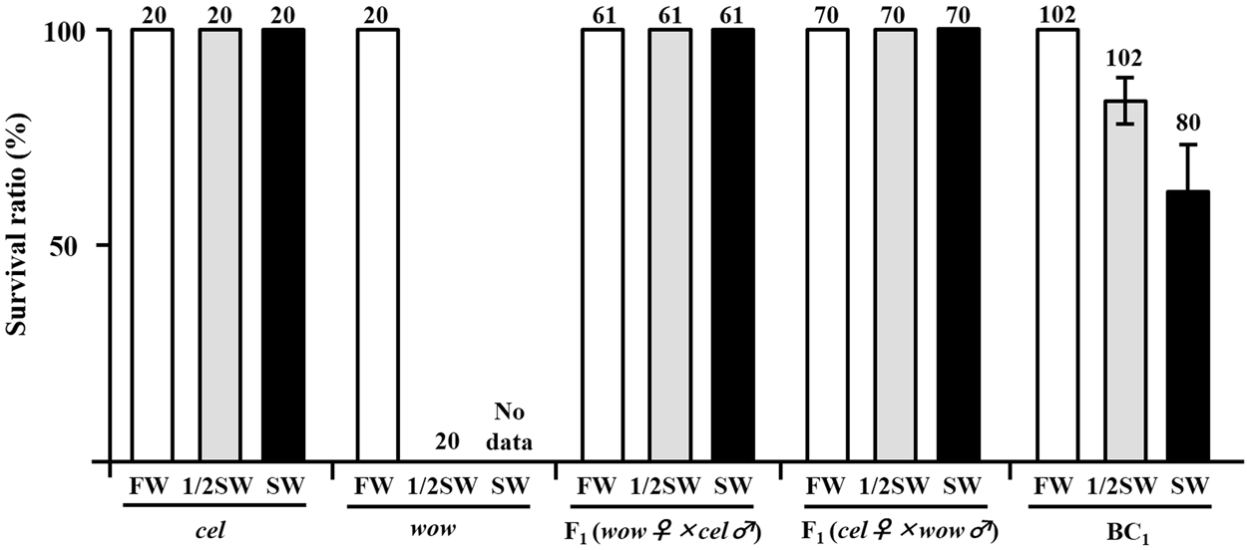
Survival of *Oryzias celebensis* and *O*. *woworae* and their F_1_ and backcross (BC) progeny after direct transfer from fresh water (FW) to FW and hypertonic half-strength sea water (SW), and from half-strength SW (1/2 SW) to full-strength SW. BC progeny were obtained by crossing F_1_ (*O*. *celebensis* females × *O*. *woworae* males) females and *O*. *woworae* males. Since all *O*. *woworae* died in half-strength SW within 1 week, no data are shown for the survival of *O*. *woworae* in SW. *cel*, *O*. *celebensis; wow*, *O*. *woworae*. The numbers above the column indicate the total number of individual in each test. Error bars mean standard deviations.

All mature *O*. *celebensis*, *O*. *luzonensis*, *O*. *pectoralis*, and *O*. *mekongensis* (N = 20 each) fish survived after direct transfer from fresh water to half-strength sea water (Fig. 1). When the fish maintained in half-strength sea water for 7 days were transferred to full-strength sea water, no fish died even after prolonged exposure to sea water for 1 month. In contrast, all *O*. *woworae* and *O*. *profundicola* individuals died within 1 week after transfer to half-strength sea water (N = 20, Figs 1 and 2). Since these two species may not be able to adjust to rapid transfer to hyperosmotic water with handling stress, we performed an additional survival test of *O*. *woworae* without the handling in hyperosmotic water (Supplemental Fig. 1). All survived individuals died in full-strength sea water, suggesting that *O*. *woworae* cannot adjust to full-strength sea water at least.

To investigate whether the difference in hyperosmotic tolerance is a genetic trait, we initially examined the F_1_ hybrids of *O*. *celebensis* and *O*. *woworae*. All F_1_ adults survived after the transfer to half-strength sea water, and no fish died even after further transfer to sea water, as observed in *O*. *celebensis*, suggesting that the hyperosmotic tolerance trait is dominant (Fig. 2). Similar results have been reported in hybrids of *Oreochromis niloticus* and *Sarotherodon galilaeus*^12^. Furthermore, we backcrossed the F_1_ *O*. *celebensis* females to *O*. *woworae* males, and 22% (22/102) of the adult backcross (BC) progeny died in half-strength sea water. When the remaining 80 BC progeny were further transferred to full-strength sea water, 48 individuals survived. Thus, the total survival ratio was 47% (48/102), and the hyperosmotic tolerant and non-tolerant phenotypes segregated essentially in a 1:1 ratio. These results suggest that the *O*. *celebensis* allele at a single major locus is responsible for the hyperosmotic tolerance but the involvement of handling stress cannot be completely excluded.

### The locus for adult hyperosmotic tolerance maps to a fused chromosome corresponding to *O*. *latipes* linkage groups 6 and 23

**Figure 3:**
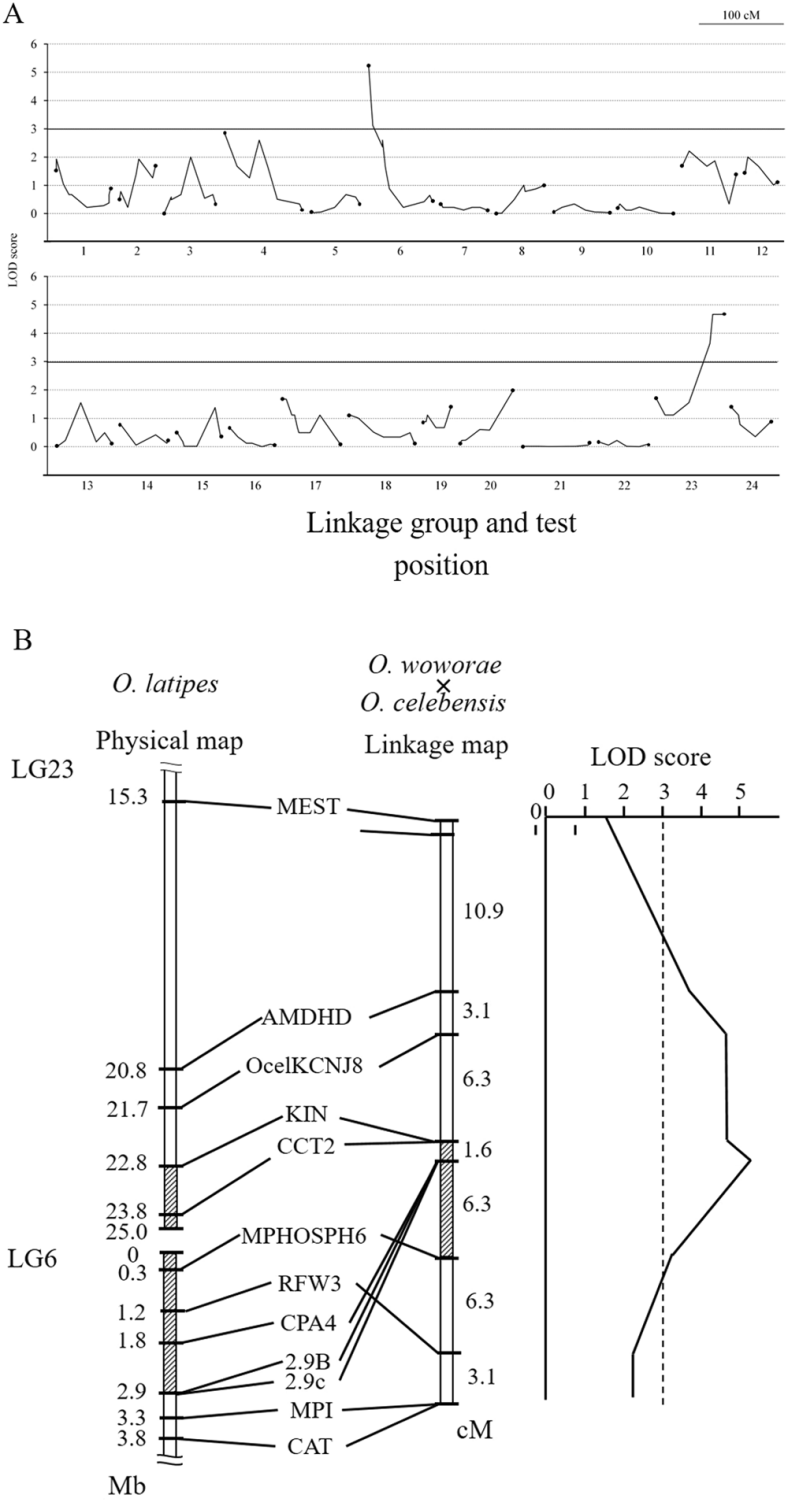
(**A**) Multipoint LOD scores for a genome-wide scan. Backcross (BC) progeny that survived or died within a 7-day exposure period (32 individuals each) in sea water were genotyped for 184 marker loci throughout the genome. Scores are plotted for marker loci spanning linkage groups (LG) 1–12 (upper tier) and 13–24 (lower tier). Relative lengths and marker distances along each LG will be reported elsewhere. Horizontal solid lines represent the threshold LOD score of 3. Closed circles at either end of a polyline indicate markers at the ends of each linkage map. (**B**) Linkage map of the hyperosmotic tolerance loci in F_1_ adults of *Oryzias woworae* × *O*. *celebensis* and physical *O*. *latipes* LG 6 and LG 23 maps. The LOD score is indicated across the top and is plotted relative to the positions of the markers shown. The distance between flanking markers is shown as the physical length (*left*) or in cM (*right)*. The shaded areas represent the region determining hyperosmotic tolerance on the genetic maps and the corresponding regions on the physical maps.

To search for the locus pertaining to adult hyperosmotic tolerance, we conducted a genome-wide linkage analysis on surviving and dead BC progeny (32 individuals each) using 211 polymorphic DNA markers. Figure 3A shows the marker positions for 24 linkage groups (LGs) and the correlation between hyperosmotic-tolerant phenotypes and DNA marker genotypes. This analysis identified eight markers significantly associated (LOD score > 3.0) with hyperosmotic tolerance phenotypes (Fig. 3B). All markers were mapped at the ends of LGs 6 and 23 in the *O*. *latipes* linkage map, which corresponds to the central region of a fused chromosome in *O*. *celebensis* and *O*. *woworae*. Three markers on LG6, CPA4, 2.9B, and 2.9c were most tightly linked (LOD = 5.24) to the hyperosmotic tolerance locus flanked by the markers CCT2 and MPHOSPH6. This region spanned approximately 4.1 Mb of the *O*. *latipes* genome sequence (http://asia.ensembl.org/index.html). These results suggest that a single locus located approximately in the 4-Mb region of the fused chromosome is responsible for the hyperosmotic tolerance of adult fish.

The corresponding regions on *O*. *latipes* LGs 6 and 23 contain 132 annotated genes in the Ensemble genome browser (http://asia.ensembl.org/index.html). Some of these candidates are members of the solute carrier and ATP-binding cassette transporter families that are functionally related to osmotic regulation. For teleosts to maintain their hydromineral balance in sea water, the flux of ions relies primarily on these epithelial transporters, including Na^+^/K^+^-ATPase, Na^+^-K^+^-2Cl^−^, and the cystic fibrosis transmembrane conductance regulator (Cl^−^ channel), in osmoregulatory organs, as has been discussed in some reviews^3,7,13^. Quantitative trait analyses for hyperosmotic tolerance (i.e. Na+/K+ -ATPase activity and blood plasma osmolality) in Arctic charr and rainbow trout have suggested that allelic variation at the Na^+^/K^+^-ATPase a1b and insulin-like growth factor 2 loci is associated with a difference in tolerance^6^, although the causal gene has not been mapped to the above-mentioned region. Nevertheless, polymorphisms of these transporters or their regulatory factors, including *cis*-regulatory elements, appear to be involved in the hyperosmotic tolerance of adult fish.

### A maternal factor essential for embryonic hyperosmotic tolerance and its genetic basis

**Figure 4:**
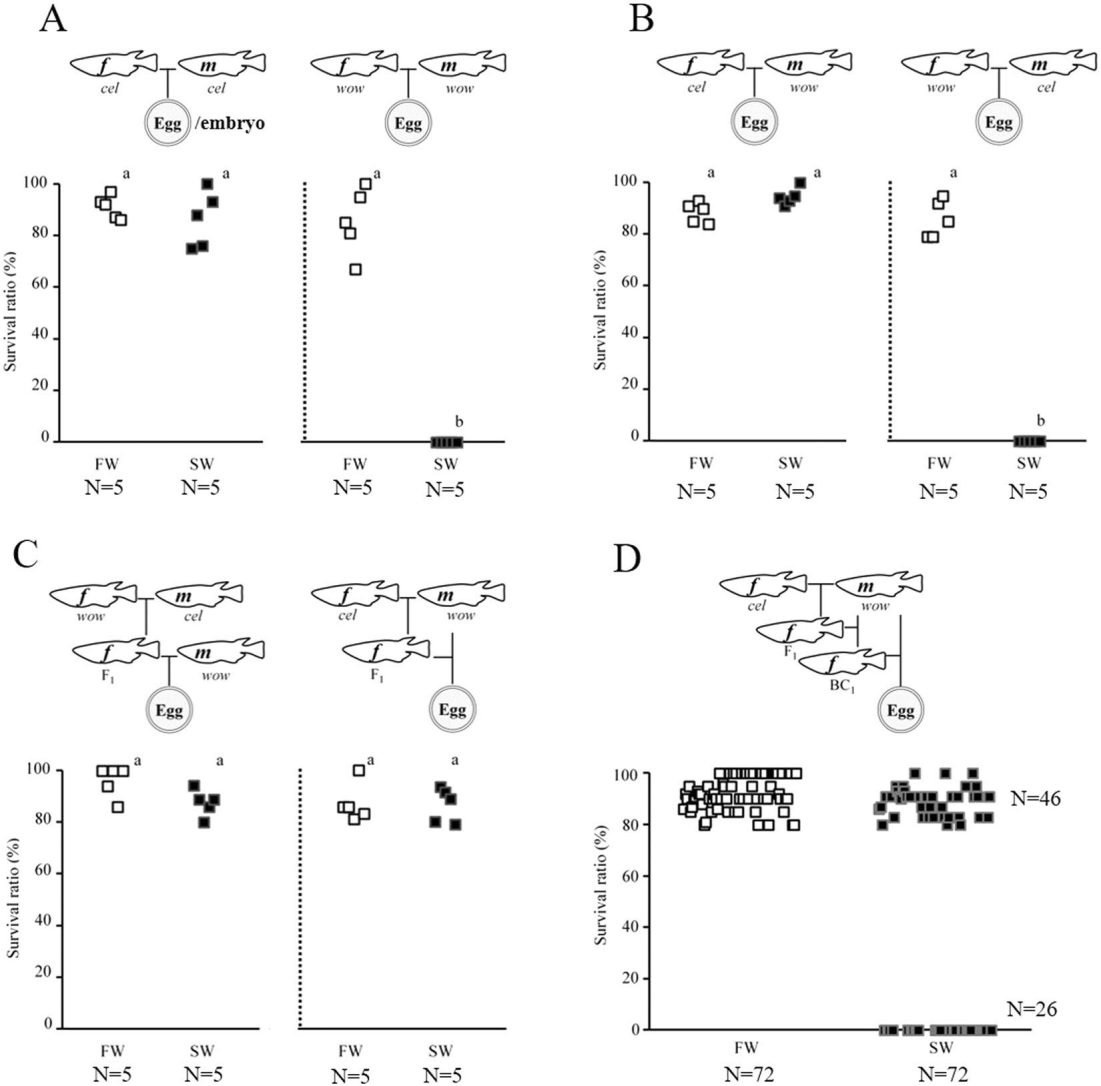
Survival tests of early embryos derived from *Oryzias celebensis* pairs (**A**) *O*. *woworae* pairs (**A**) *O*. *celebensis* females with *O*. *woworae* males (**B**), *O*. *woworae* females with *O*. *celebensis* males (**B**) the F_1_ hybrid females with *O*. *woworae* males (**C**) and backcross (BC) females with *O*. *woworae* males (**D**). Mating schemes are shown above each graph. Eggs, spawned and fertilised in fresh water, were transferred to fresh water (FW) or sea water (SW) after 1 h (1 to 2 cell stage). Survival ratios 24 h after fertilisation were calculated at least 20 eggs/embryos from five pairs (**A**–**C**) or 72 pairs (**D**). Open and closed squares indicate survival ratios of embryos from each female. Groups with different letters in each graph are significantly (P < 0.05) different. *f*, female; *m*, male; *cel*, *O*. *celebensis*; *wow*, *O*. *woworae*.

After transfer to sea water at the 1 to 2 cell stage, all fertilised eggs/embryos derived from five pairs of *O*. *celebensis* developed normally (Fig. 4A), similarly to some euryhaline *Oryzias* species^10^. These embryos that developed to the somite stage in fresh water (24 h after fertilisation) were also able to survive in sea water, as in the case of adults. In contrast, all embryos derived from five pairs of *O*. *woworae* died (cleavage ceased) within 10 h of fertilisation when reared in sea water, but survived and developed normally in fresh water. To elucidate the difference in hyperosmotic tolerance, we conducted an additional survival test on the hybrid embryos. All embryos derived from *O*. *celebensis* females and *O*. *woworae* males developed in sea water similarly to the embryos from *O*. *celebensis* pairs (Fig. 4B). Interestingly, all embryos derived from *O*. *woworae* females and *O*. *celebensis* males died in sea water similarly to the embryos from the *O*. *woworae* pair. These results suggest that a maternal factor(s) derived from *O*. *celebensis* eggs is responsible for hyperosmotic tolerance during the cleavage stage, since zygotic gene expression has been reported to begin at the early blastula stage in *Oryzias* species^14^. In contrast, the hybrid late embryos were able to survive in sea water, as in the case of adults, partially by counterbalancing the undeveloped osmoregulatory gills with the numerous ionocytes in the body and/or yolk sac^7,15,16^. To understand the genetic inheritance of the maternal factor(s) related to embryonic hyperosmotic tolerance, we examined the embryos derived from F_1_ hybrid females and *O. woworae* males (Fig. 4C). These fertilised eggs developed in sea water similarly to those in fresh water and to those from the *O*. *celebensis* pairs, suggesting that the trait controlled by the maternal hyperosmotic tolerance factor(s) is dominant. Furthermore, the embryos from approximately 60% (46/72) of BC females developed in sea water similarly to those from the *O*. *celebensis* pair, whereas the embryos from the remaining 40% (26/72) of BC females died in sea water (Fig. 4D). These results suggest that one or a few major loci are responsible for the maternal hyperosmotic tolerance factor.

### A maternal factor linked to embryonic hyperosmotic tolerance maps to *O*. *latipes* LG 17

**Figure 5:**
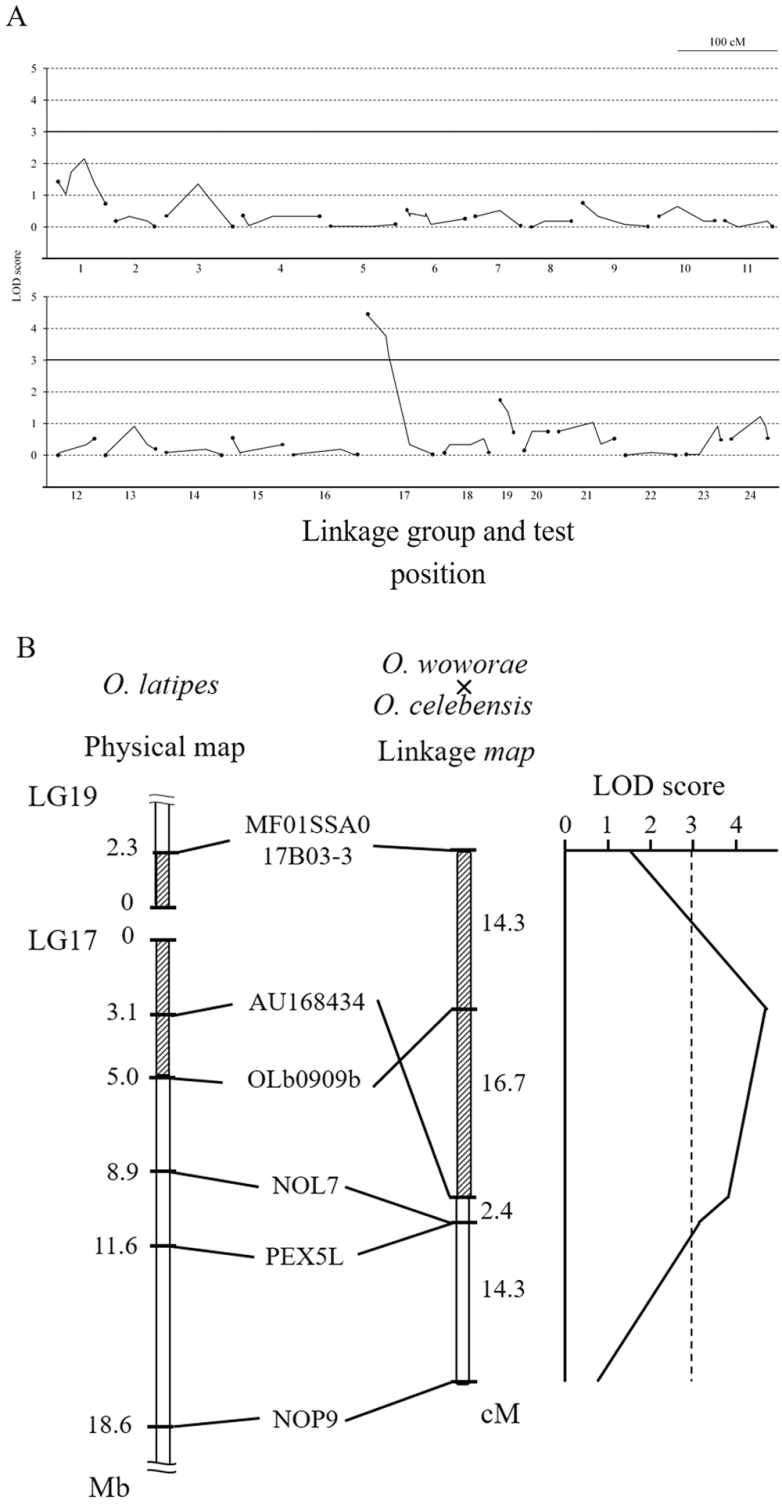
(**A**) Multipoint LOD scores for the genome-wide scan. Backcross (BC) females (26 individuals spawning hyperosmotic tolerant eggs and 16 spawning non-hyperosmotic tolerant eggs) were genotyped for 107 marker loci throughout the genome. The horizontal solid lines represent the threshold LOD score of 3. Closed circles at either ends of a polyline indicate markers at the ends of each linkage map. (**B**) Linkage map of the embryonic hyperosmotic tolerance loci in F_1_ adults of *Oryzias woworae* × *O*. *celebensis* and physical *O*. *latipes* LG 9 and LG 17 maps. The LOD score is indicated across the top and is plotted relative to the positions of the markers shown. The distance between flanking markers is shown as a physical length (*left*) or in cM (*right)*. The shaded areas represent the region determining embryonic hyperosmotic tolerance on the genetic maps and the corresponding regions on the physical maps.

To search for a locus linked to the maternal factor(s) determining embryonic hyperosmotic tolerance, 42 BC females (26 females spawning hyperosmotic-tolerant eggs and 16 females spawning non-tolerant eggs) were screened for 107 polymorphic DNA markers. Figure 5A indicates a correlation between a maternal hyperosmotic tolerance factor(s) (i.e. hyperosmotic tolerance of eggs) and genotypes of the BC females. One marker on LG 17 was most tightly linked to the causal locus. Further linkage analyses revealed that four markers, AU168434, OLb0909b, NOL7, and PEX5L, were significantly linked to the egg hyperosmotic tolerance phenotypes (LOD score > 3.0). All markers mapped to the middle region of a fused chromosome in *O*. *celebensis* and *O*. *woworae* corresponding to *O*. *latipes* LGs 17 and 19 (Fig. 5B). The marker most tightly linked to the locus responsible for the maternal hyperosmotic tolerance factor(s) (i.e. hyperosmotic-tolerant eggs) was OLb0909b (LOD score = 4.42), which was located between MF01SSA017B03-3 and AU168434, spanning approximately 7.3 Mb of the *O*. *latipes* genome sequence (http://asia.ensembl.org/index.html). These results suggest that a single locus on a distinct chromosome is responsible for the hyperosmotic tolerance of embryos during early developmental stages, namely the maternal hyperosmotic tolerance factor(s).

The corresponding region on *O*. *latipes* LG 17 and 19 includes 187 annotated genes. Among these candidates, phosphofructokinase and acyl-CoA dehydrogenase are implicated in basic metabolism (http://asia.ensembl.org/index.html). After exposure of fertilised eggs and early embryos to a hyperosmotic environment before the organisation of osmoregulatory epithelia, the synthesis/activation of these possible maternal enzymes might be involved in the prevention of dehydration by producing free amino acids as intracellular osmolytes, such as has been observed in osmoconforming marine invertebrates^17^ and in developing seabass larvae^18–20^.

Several BC individuals, which had no candidate regions from *O*. *celebensis* mentioned above, survived in sea water or spawned hyperosmotic-tolerant eggs, suggesting the presence of minor modifiers that affect tolerance at the adult or early embryonic stages. To remove these effects, we are currently establishing congeneric strains with the candidate region of *O*. *celebensis* in the genetic background of *O*. *woworae* by repeated backcrossing of the BC individuals to *O*. *woworae*.

## Conclusions

In this study, we discovered two major genomic loci discretely responsible for hyperosmotic tolerance during the adult and early embryonic stages in teleosts, suggesting that distinct mechanisms play critical roles in hyperosmotic tolerance during their life history. Our findings may provide a basis for further clarifying the essential regulatory mechanisms of osmotic tolerance at the molecular level not only in adult but also early embryonic teleosts. The osmoregulatory mechanisms suggested here for adults are observed in some evolutionarily advanced taxa like vertebrates, whereas most marine invertebrates are osmoconformers requiring intracellular regulation, similar to that suggested here for early embryos^21^. Thus, the present genetic study of the determinants of osmotic tolerance may also provide insight into the ontogeny and phylogeny related to adjustment strategies.

## Methods

### Fishes

In this study, the care and treatment of animals were carried out under protocols approved by the Animal Care and Use Committee of Niigata University. All experiments were performed in accordance with relevant guidelines and regulations of the Animal Care and Use Committee of Niigata University. *Oryzias celebensis*, *O*. *luzonensis*, *O*. *pectoralis*, *O*. *mekongensis*, and *O*. *profundicola* were supplied from laboratory stocks maintained at Niigata University, a sub-centre of the National Bio Resource Project (Medaka) in Japan, and *Oryzias woworae* were purchased from a commercial source (Charm, Gunma, Japan; Supplemental Fig. 1). For the genetic analysis of hyperosmotic tolerance in adult fish and early embryos, F_1_ progeny were produced by mating *O*. *celebensis* females with *O*. *woworae* males or *O*. *woworae* females with *O*. *celebensis* males. Backcross (BC) progeny were produced by mating F_1_ (*O*. *celebensis* females × *O*. *woworae* males) females with *O*. *woworae* males. All fish were reared in aged tap water at 27 °C ± 2 °C under a 14:10 h light: dark cycle.

### Survival test of adult fish in hyperosmotic water

Individuals of the five species (*O*. *luzonensis*, *O*. *pectoralis*, *O*. *profundicola*, *O*. *celebensis*, and *O*. *woworae*), F_1_ progeny, and BC progeny used in the test were mature (>20 mm in length, N = 20) and had been acclimated to fresh water for at least 2 weeks. Ten fish from each group were directly transferred into a plastic aquarium filled with 5 L of fresh water (control group) or half-strength artificial sea water (test group) (15 ppt Sealife, Marinetech, Tokyo, Japan). Survival was monitored daily for 7 days. Death was defined as the termination of opercular movement^8^. Fish that survived in half-strength sea water for 7 days were transferred to full-strength (30 ppt) sea water, and the survival of these fish was similarly monitored.

### Survival test of fertilised eggs/early embryos in sea water

Eggs were spawned and fertilised in fresh water and then directly transferred to experimental aquaria containing either fresh or sea water (30 ppt) after 1 h (1 to 2 cell stage). Eggs were incubated separately using 24-well culture plates at 27 °C ± 2 °C. Embryo survival was monitored after 24 h. Survival ratios were calculated from at least 20 eggs per female.

### Genotyping and linkage analysis

Total genomic DNA was extracted from a caudal fin clip or tail muscle sample using a standard method of proteinase K digestion in lysis buffer, followed by a phenol:chloroform extraction^22^. To search for hyperosmotic tolerance-linked markers, we used expressed sequence tags (ESTs) established for *O*. *latipes*, which were amplified using previously published primers designed for *O*. *latipes*^23^. PCR genotyping for 15 loci (MEST, AMDHD, OcelKCNJ8, KIN, CCT2, CPA4, 2.9B, 2.9c, MPHOSPH6, RFWD3, MPI, CAT, NOL7, PEX5L, and NOP9) was conducted using primers designed based on the EST sequences (http://mbase.bioweb.ne.jp/~dclust/medaka_top.html) and the draft genome sequence of *O*. *latipes*^24^. PCR amplification of each marker was performed as follows: 4 min at 95 °C, followed by 12 cycles of 30 s at 95 °C, 30 s at 65–56 °C, and 2 min at 72 °C, and 22 cycles of 30 s at 95 °C, 30 s at 53 °C, and 2 min at 72 °C, with a final elongation step of 2 min at 72 °C. Polymorphisms between *O*. *celebensis* and *O*. *woworae* were identified by length differences in the PCR products, restriction-fragment length polymorphisms, or heteroduplex polymorphisms using discontinuous polyacrylamide gels with a 4% stacking gel and a 9% separating gel^25–27^. Marker names, assigned LGs, and primer sequences are listed in Supplemental Table 1. A logarithm of the odds (LOD) score of 3.0 was adopted as an indication of potential hyperosmotic tolerance-linked markers.

### Statistical analysis

The effect of salinity on the survival rates of early embryos was initially tested with a two-way analysis of variance (ANOVA) to examine whether there was an interaction effect between strain and salinity. If the two-way ANOVA detected a significant interaction, the survival rate of each strain was further compared with separate one-way ANOVAs followed by a Tukey-Kramer post-hoc test for each salinity level. The probability for establishing statistical significance was P < 0.05.

### Availability of data and materials

All data generated or analysed during this study are included in this published article (and its Supplementary Information files).

## Electronic supplementary material

Supplementary dataset
