# Supplementary material for: Hyperosmotic tolerance of adult fish and early embryos are determined by discrete, single loci in the genus Oryzias
Source: Sci Rep. 2018 May 2;8:6897. doi: 10.1038/s41598-018-24621-7 (PMC5932013; doi:10.1038/s41598-018-24621-7)
Supplement: Supplementary file 1 — Supplementary dataset [file 41598_2018_24621_MOESM1_ESM.pdf]

Hyperosmotic tolerance of adult fish and early embryos are determined by discrete, single loci in the genus *Oryzias*

Taijun Myosho[1,2], Hideya Takahashi[2,3], Kento Yoshida[2], Tadashi Sato[2], Satoshi Hamaguchi[2], Tatsuya Sakamoto[3], and Mitsuru Sakaizumi[2]

[1]Laboratory of Molecular Reproductive Biology, Institute for Environmental Science, University of Shizuoka, Shizuoka, Shizuoka 422-8526, Japan

[2]Department of Environmental Science, Institute of Science and Technology, Niigata University, Ikarashi, Niigata 950-2181, Japan

[3]Ushimado Marine Institute, Faculty of Science, Okayama University, Setouchi, Okayama 701-4303, Japan

Correspondence and requests for materials should be addressed to T.M.

(taijun@u-shizuoka-ken.ac.jp)

Supplemental Figure 1

Additional survival tests of *Oryzias woworae* after by gradual and indirect transition of fresh water (FW) in the aquaria to hypertonic half-strength sea water (1/2 SW), and transition of 1/2 SW to full-strength sea water (SW). The transitions were completed about 5 h. In both of the two tests, thirty percent individuals of *O. woworae* survived in 1/2 SW, and all individuals died in SW. The numbers above the column indicate the total number of individuals in each test.

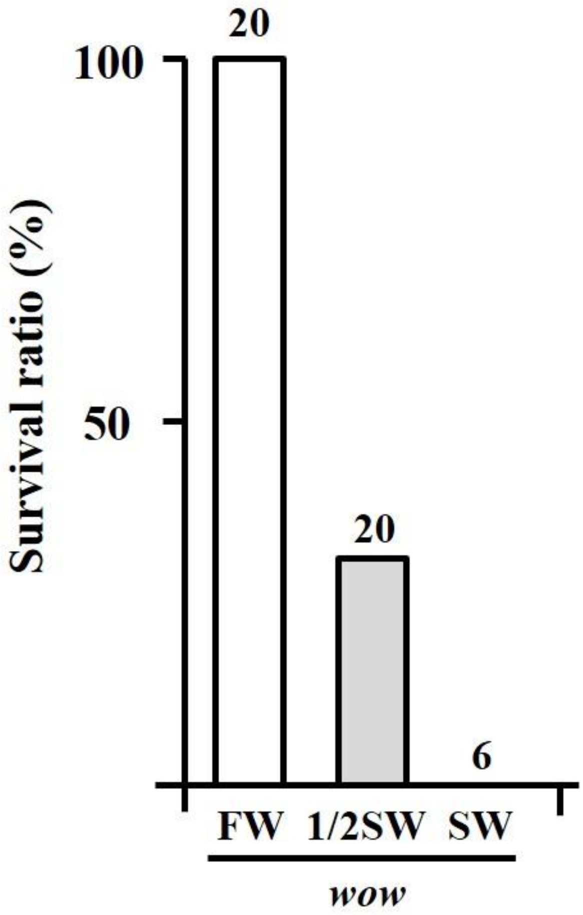

Supplemental Figure 2

The 50% majority-rule consensus tree of a maximum likelihood of the *COI* region

The *COI* sequence of the commercial *Oryzias woworae* in this study completely matched with two native *O. woworae* sequences. Furthermore, the commercial *O. woworae* made a clade with two local *O. woworae.* The *COI* sequence was determined by PCR-direct sequence using previously designed primers[1]. The *COI* sequences were preliminarily aligned in MEGA 5.1 using ClustalW[2]. The phylogenetic relationships were analyzed by maximum likelihood. The GenBank accession numbers of the DNA sequences are as follows: *COI* sequences, JX311925- JX311944.

1. Parenti, L. R., Hadiaty, R.K., Lumbantobing, D. & Herder, F. Two new ricefishes of the genus Oryzias (Atherinomorpha: Beloniformes: Adrianichthyidae) augment the endemic freshwater fish fauna of southeastern Sulawesi, Indonesia. *Copeia* **3**, 403-414 (2013).
2. Tamura, K. *et al*. MEGA5: molecular evolutionary genetics analysis using maximum likelihood, evolutionary distance, and maximum parsimony methods. *Mol biol evol.* **28**, 2731-2739 (2011).

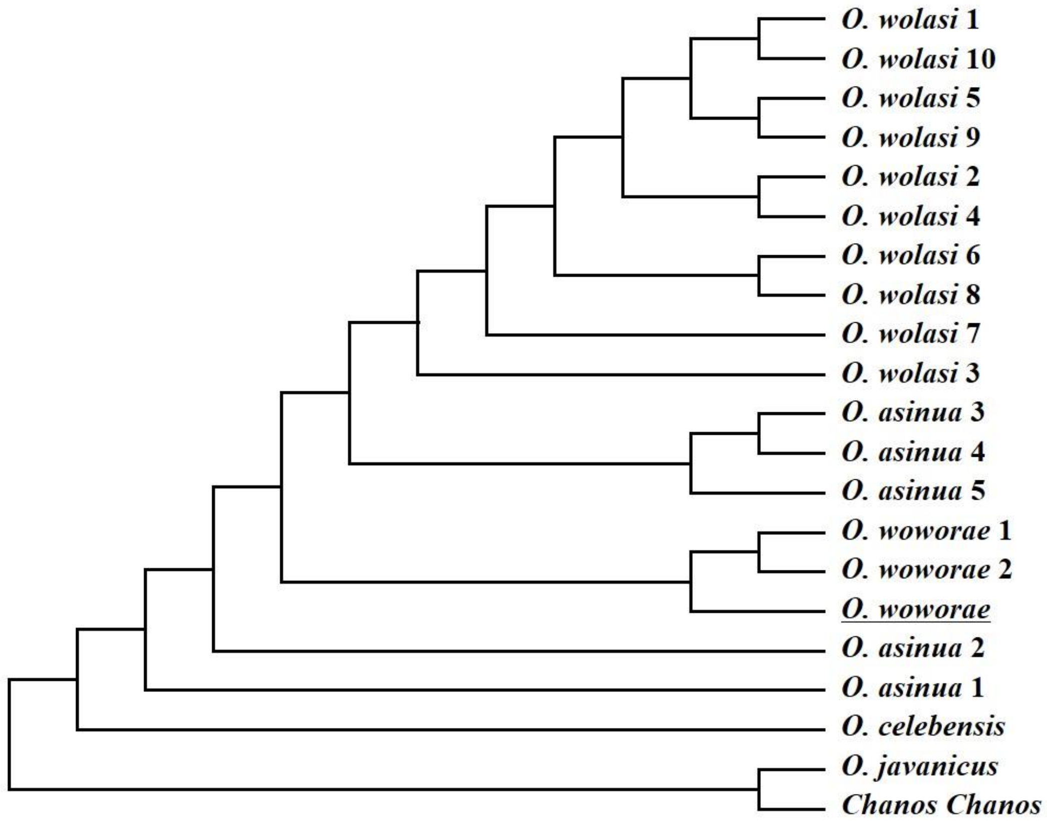
O. wolasi 1
O. wolasi 10
O. wolasi 5
O. wolasi 9
O. wolasi 2
O. wolasi 4
O. wolasi 6
O. wolasi 8
O. wolasi 7
O. wolasi 3
O. asinua 3
O. asinua 4
O. asinua 5
O. woworae 1
O. woworae 2
O. woworae
O. asinua 2
O. asinua 1
O. celebensis
O. javanicus
Chanos Chanos

Supplementary Table 1

EST an STS primers for constructing linkage map of embryonic and adult hyperosmotic tolerance

LG	Name	forward	reverse
6	MPHOSPH	TCCAAGAATCTGCTGCGCATGAAG	CCTCCTCTCGTCTTCCTCCAGC
	RFWD3	AGGTCTCTGGAGCAGGAACAG	CCAGCACCGCCTTGGAGAAG
	CPA4	ACAGCTGGATGGACACTCTG	GGGTTGGCCAGAATCAGCATG
	2.9B	AGCTTTCCAGAACTGGCTGA	TGGCGTCCATTTTGAAGATT
	2.9c	GGCCATCAAGTTTCTTTCCA	GCTACACGCATCAAAAGCAC
	MPI	AAGAAGTGCTTCACCAGGATG	CACACGGGAAGATTTTGGAG
	CAT	ACATGGTGTGGGACTTCTGG	AGCCGTAGCCGTTCATGTGG
17	AU168434	TGGAGCTTGGACCAAAGCCAGATGTTGC	GGAACTGGAGTGGACTCACACGCAGA
	OLb0909b	AGGTGCCGAGCTTGTTGAAGCG	ATCTGCACCGGGACGATTTGGC
	NOL7	AGGAGTTGTTTCAAGAGCAGAAG	CGTCGTCACCGTGTAGTTTC
	PEX5L	GCTCTGGACTTGGTGGACCTC	AGCAGCTCGCTGGATGAGTG
	NOP9	TCTTGGCTGCAGGTCACATG	CAGGCTGCTGAGGAGAAGCGA
19	MF01SSA017B03-3	GGTGACTGGGTTTCAGCCGCAGGATGT	CCCCACGTTTTCCGGGGTTTCATAGCGA
23	MEST	TCTGGTTTCCCATGACTACGG	GTCCACATGTCCCAGAACTC
	AMDHD1	CCCATCAACATCTCCTCCAC	TAGGCAGCGTTGATGGTGGC
	OcelKCNJ8	TCATCATCTGCCACGTCATAG	GGCTTCTCGTCCAGCTCTC
	KIN	TGTCACTGCATGTCCGAGTC	TTCTTCTTCCTCGCCAGCTCC
	CCT2	GATCTACAACTACCCGGAGCAG	CTTGCCTGGCGTCCTGTTGG
